# A Rare Case of Spindle Cell Sarcoma With Rare Asymptomatic Cerebellar Metastasis

**DOI:** 10.7759/cureus.54766

**Published:** 2024-02-23

**Authors:** Candace Miyaki, Kathie Wu, David Nye, Nadia Ramdin

**Affiliations:** 1 Internal Medicine, Geisinger Medical Center, Danville, USA; 2 Palliative Care, Penn State Health, Hershey, USA; 3 Oncology, Geisinger Medical Center, Danville, USA

**Keywords:** brain metastasis, pulmonary artery tumor thrombus, soft-tissue sarcoma, pulmonary artery tumor, spindle cell sarcoma

## Abstract

Sarcomas are one of the rarest cancers, occurring in less than 1% of all adult malignancies. Spindle cell sarcomas are one subset of soft tissue sarcomas that are even less commonly reported in the literature due to the scarcity of cases, especially with the presence of brain metastases. We present a case of an adult male who presented with non-specific exertional dyspnea and chest pain, which was found to have spindle cell sarcoma with cerebellar metastasis.

## Introduction

Soft tissue sarcomas are of mesodermal origin and the classification of these neoplasms is based on the presumptive tissue of origin [1]. Common sites of occurrence include the head and neck, retroperitoneum, and extremities [2]. Sarcomas tend to recur locally but have the potential to metastasize to distant sites, most frequently to the lungs [3]. The risk of metastasis has been associated with higher-grade malignancy and the size of the primary tumor. In rare cases, spindle cell sarcomas have also been reported to metastasize to the brain [4].

## Case presentation

**Figure 1:**
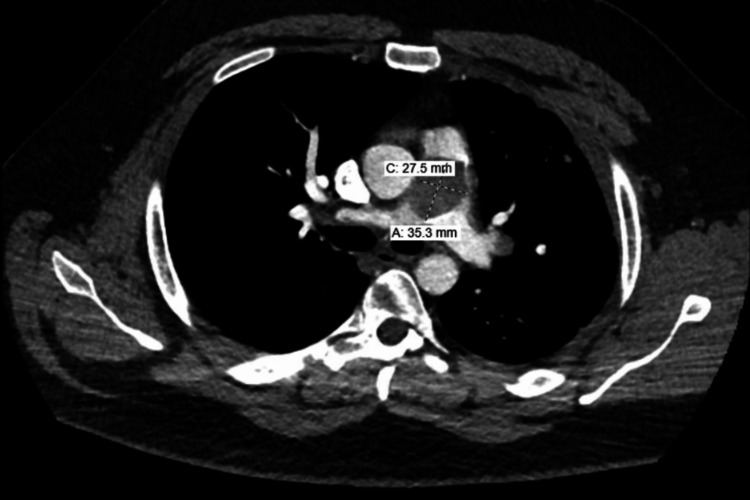
Pulmonary mass on CT imaging

**Figure 2:**
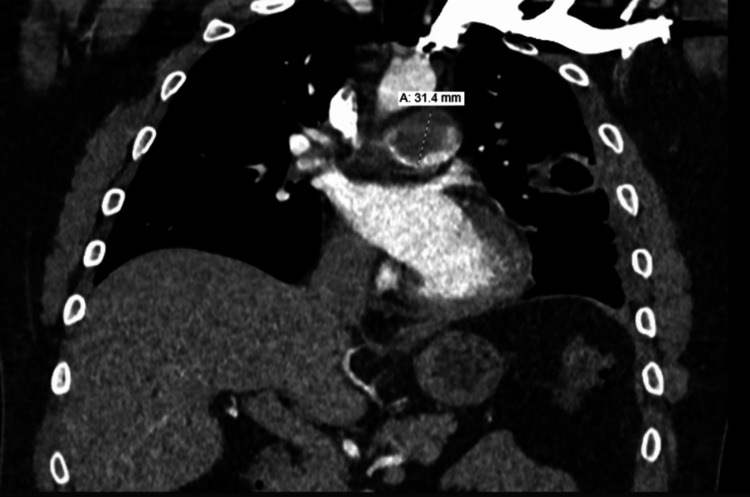
Coronal view of the pulmonary mass

**Figure 3:**
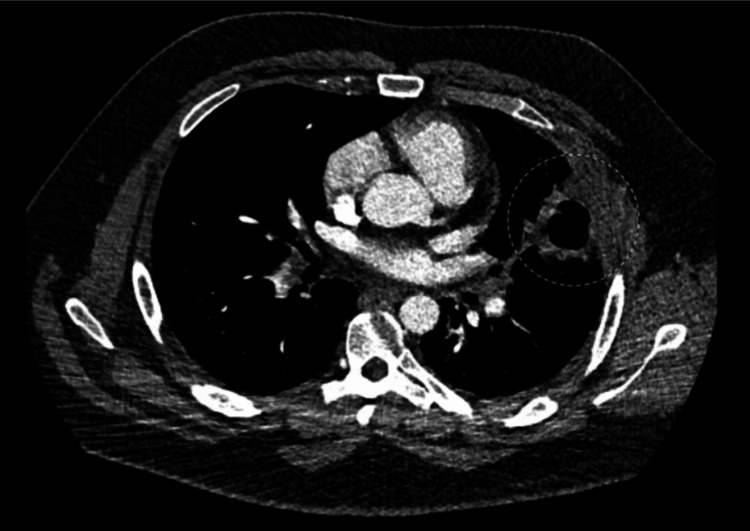
Cavitary lesion in the left lingula

**Figure 4:**
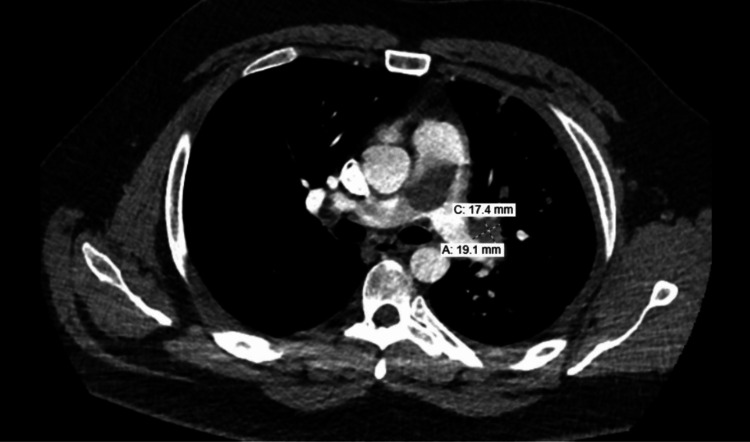
Filling defects distal to the pulmonary mass

**Figure 5:**
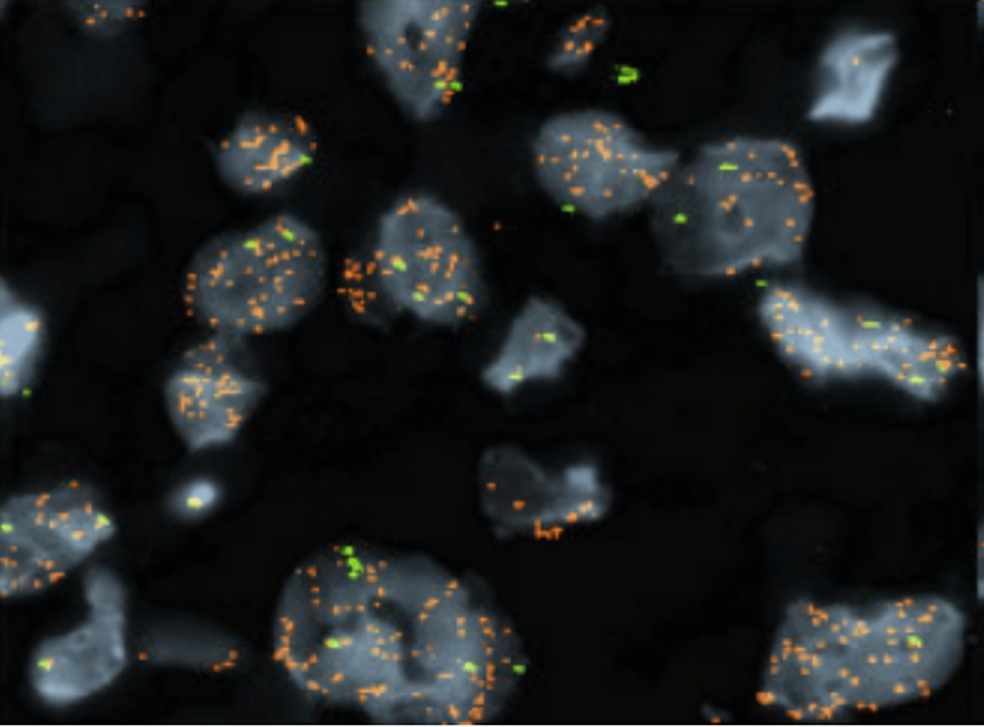
Positive MDM2 FISH assay FISH: fluorescence in situ hybridization

A 30-year-old male with a 12-pack year smoking history presented to primary care with a new heart murmur. He was scheduled for an echocardiogram, which showed right ventricular overload with dilation (>4.2 cm), decreased right ventricular systolic function, and an enlarged right atrium. About eight days later, the patient presented to the emergency department with reports of chest pain and exertional dyspnea. CT imaging showed a 2.9 x 3.4 cm oblong filling defect within the pulmonary artery (Figures 1, 2) with scattered filling defects in the right pulmonary artery, multiple nodular opacities in the left upper lobe (largest 4.8 cm) with a few peripheral nodular opacities in the bilateral lower lobes, a 4.9 x 4.0 cm cavitary lesion in the lingula that extended to the adjacent pleural surface (Figure 3), right heart strain RV/LV ratio of 1.2, and hilar lymphadenopathy. Due to difficulty distinguishing between mass and emboli, further imaging with MRI was obtained. MRI showed contrast enhancement in the main pulmonary artery, hilar soft tissues, lingula cavitary lesion, and left upper and lower lobe nodules. It also showed a tumor thrombus of the main pulmonary artery that extended to occlude the lingular pulmonary artery (Figure 4). CT-guided biopsy of a left lung nodule was done. Pathology showed a malignant spindle cell neoplasm positive for SMARCA2&4 and BAP1 (intact expression); negative for epithelial markers (MOC31, CK7, CK5/6), TTF-1, p40, calretinin, CD31, ERG, desmin, caldesmon, and S100; the proliferative index (MIB-1) was estimated at 15-20%. The MDM2 gene was amplified by fluorescence in situ hybridization (FISH) assay (Figure 5). Pathological origin showed intimal sarcoma vs. liposarcoma, favoring the former. Full body scans were done for staging. No evidence of extending disease was found on the CT abdomen and pelvis. Although the patient had no neurological evidence of disease, an MRI brain was done for complete evaluation, which showed an 8 mm enhancing lesion in the right cerebellum concerning for metastasis. The patient was noted not to be a good surgical candidate due to the degree of his high risk of decompensation, so was medically managed. He was started on apixaban for the tumor thrombus, a regimen of doxorubicin, mesna, and ifosfamide for his spindle cell lesions, and radiation treatment for his solitary cerebellar lesion. During his treatment course, his apixaban was held when platelets dropped below 30,000 due to the risk of spontaneous hemorrhage. Four months later, the patient returned to the emergency room again with concerns of worsening shortness of breath and chest pain. Repeat CT imaging at that time showed an increased burden of pulmonary emboli in the right pulmonary artery, decreased cavitary lesion, increased size of left lung nodules, additional left lung nodules, decreased mass in the main and right pulmonary arteries, and right heart strain. The patient was deemed a poor candidate for thrombectomy, so the decision was made again to medically manage. Given the presence of worsening clot burden, his anticoagulation was transitioned to weight-based enoxaparin.

## Discussion

Sarcomas often present with nonspecific symptoms including shortness of breath, cough, lightheadedness, hemoptysis, and chest pain [5]. In addition, they are often painless, which further contributes to delay in diagnosis [3]. These tumors are known to have poor prognoses, with average survival from initial diagnosis being a matter of months [6]. Given the aggressive nature of these malignancies, local recurrence and metastasis often occur [3,7]. The most common sites of metastases are the lungs, liver, and bones, but brain metastases have also been reported in 1-8% of cases [4,8]. Some studies suggest that the presence of brain metastases may even be as low as 0.7% [4]. One retrospective study with 112 individuals with malignant sarcomas showed that most primary sarcomas originated in the extremities with initial metastases to the pulmonary system. In the late stage of the disease, brain metastases did occur, mostly affecting the frontal lobe (40%), and least commonly, the cerebellum (8%). Of those 112 individuals, only 8% had spindle cell sarcomas. In fact, undifferentiated sarcomas, as a group, showed low metastatic rates compared to other soft tissue sarcomas such as alveolar or osteosarcoma [9]. It was shown that brain metastases represent late-stage disease with a median survival of five months after occurrence [10].

The median age of spindle cell sarcoma diagnosis is 57 [1], but our patient was much younger at the time of diagnosis. Despite the earlier age of presentation, he had evidence of late-stage disease with distant metastases to the distal lungs and the cerebellum. Due to the patient not being a good candidate for surgical intervention or embolectomy, he was treated medically. Although he showed relative disease stability with chemotherapy four months after initial diagnosis, the complications from distal tumor emboli required subsequent hospital admission. It remains unclear whether he truly failed apixaban therapy, given his doses were held during periods of thrombocytopenia but given the presence of a worsening clot burden on subsequent imaging, it was warranted to transition to a weight-based therapy to ensure proper treatment.

## Conclusions

The presentation and management of spindle cell sarcomas, particularly in cases with brain metastases, remain scarce in literature given the low incidence of disease. Improvement of this data could lead to better screening when staging these malignancies. Although spindle cell sarcomas overall have low metastatic potential, this case shows that patients with spindle cell sarcomas should undergo brain imaging as part of the initial staging workup despite showing no neurological deficits. Despite their rarity, sarcomas should not be overlooked as a differential, especially given the nonspecific symptoms and mimicry of other disease processes on initial presentation.
